# Delivered dose of renal replacement therapy and mortality in critically ill patients with acute kidney injury

**DOI:** 10.1186/cc7784

**Published:** 2009-04-15

**Authors:** Sergio Vesconi, Dinna N Cruz, Roberto Fumagalli, Detlef Kindgen-Milles, Gianpaola Monti, Anibal Marinho, Filippo Mariano, Marco Formica, Mariano Marchesi, Robert René, Sergio Livigni, Claudio Ronco, the DOse REsponse Multicentre International collaborative Initiative (DO-RE-MI Study Group)

**Affiliations:** 1 Department of Anesthesiology and Intensive Care, Hospital Niguarda, Piazza dell'Ospedale Maggiore 3, 20162, Milan, Italy; 2 Department of Nephrology, Dialysis and Transplantation, St Bortolo Hospital, Viale Rodolfi 37, 36100 Vicenza, Italy; 3 Department of Anaesthesiology and Intensive Care I, St Gerardo dei Tintori Hospital, Via giovanni Pergolesi 33, 20100 Monza, Italy; 4 Anesthesiology Clinic, University of Düsseldorf, Moorenstrasse 5, 40225 Germany; 5 Hospital Center of Porto, Alameda do Prof. Hernâni Monteiro, 4200 Paranhos, Porto, Portugal; 6 Nephrology and Dialysis Unit, CTO Hospital, Via Gianfranco Zuretti 29, 10126 Turin, Italy; 7 Department of Nephrology, Hospital Santa Croce e Carle, Via Michele Coppino 26, 12100 Cuneo, Italy; 8 Department of Anaesthesiology and Intensive Care, Riuniti di Bergamo Hospital, Via Tito Livio 2, 24123 Bergamo, Italy; 9 Intensive Care Unit, University of Poitiers, 2, rue de la Miletrie, 86021, Poitiers, France; 10 Department of Intensive Care, Giovanni Bosco Hospital, Piazza Del Donatore di Sangue 3, 10154 Torino, Italy

## Abstract

**Introduction:**

The optimal dialysis dose for the treatment of acute kidney injury (AKI) is controversial. We sought to evaluate the relationship between renal replacement therapy (RRT) dose and outcome.

**Methods:**

We performed a prospective multicentre observational study in 30 intensive care units (ICUs) in eight countries from June 2005 to December 2007. Delivered RRT dose was calculated in patients treated exclusively with either continuous RRT (CRRT) or intermittent RRT (IRRT) during their ICU stay. Dose was categorised into more-intensive (CRRT ≥ 35 ml/kg/hour, IRRT ≥ 6 sessions/week) or less-intensive (CRRT < 35 ml/kg/hour, IRRT < 6 sessions/week). The main outcome measures were ICU mortality, ICU length of stay and duration of mechanical ventilation.

**Results:**

Of 15,200 critically ill patients admitted during the study period, 553 AKI patients were treated with RRT, including 338 who received CRRT only and 87 who received IRRT only. For CRRT, the median delivered dose was 27.1 ml/kg/hour (interquartile range (IQR) = 22.1 to 33.9). For IRRT, the median dose was 7 sessions/week (IQR = 5 to 7). Only 22% of CRRT patients and 64% of IRRT patients received a more-intensive dose. Crude ICU mortality among CRRT patients were 60.8% vs. 52.5% (more-intensive vs. less-intensive groups, respectively). In IRRT, this was 23.6 vs. 19.4%, respectively. On multivariable analysis, there was no significant association between RRT dose and ICU mortality (Odds ratio (OR) more-intensive vs. less-intensive: CRRT OR = 1.21, 95% confidence interval (CI) = 0.66 to 2.21; IRRT OR = 1.50, 95% CI = 0.48 to 4.67). Among survivors, shorter ICU stay and duration of mechanical ventilation were observed in the more-intensive RRT groups (more-intensive vs. less-intensive for all: CRRT (median): 15 (IQR = 8 to 26) vs. 19.5 (IQR = 12 to 33.5) ICU days, *P *= 0.063; 7 (IQR = 4 to 17) vs. 14 (IQR = 5 to 24) ventilation days, *P *= 0.031; IRRT: 8 (IQR = 5.5 to 14) vs. 18 (IQR = 13 to 35) ICU days, *P *= 0.008; 2.5 (IQR = 0 to 10) vs. 12 (IQR = 3 to 24) ventilation days, *P *= 0.026).

**Conclusions:**

After adjustment for multiple variables, these data provide no evidence for a survival benefit afforded by higher dose RRT. However, more-intensive RRT was associated with a favourable effect on ICU stay and duration of mechanical ventilation among survivors. This result warrants further exploration.

**Trial Registration:**

Cochrane Renal Group (CRG110600093).

## Introduction

Acute kidney injury (AKI) requiring renal replacement therapy (RRT) occurs in 5 to 6% of critically ill patients and is associated with high mortality and significant health resource utilization [1-3]. Controversy exists as to what constitutes optimal RRT in this setting. There are several modifiable factors in the delivery of RRT which may potentially influence patient outcome, including RRT modality (continuous or intermittent), solute removal mechanisms (convection, diffusion, adsorption or combination), timing of initiation and dose of treatment. The relationship between patient outcome and treatment dose was first introduced in a landmark study where patients randomised to post-dilution continuous veno-venous haemofiltration (CVVH) at a dose of 35 ml/kg/hour or above had improved survival compared with those randomised to 20 ml/kg/hour [4]. Since then, this issue has been explored in other studies with conflicting results [5-9]. The Acute Dialysis Quality Initiative recommends a higher dose in the absence of definitive data, particularly in septic patients [10,11]. However, practice surveys suggest that this threshold dose has not been widely adopted into current intensive care units (ICU) practice [12,13].

We performed a prospective European multicentre observational cohort study to evaluate the prescription and actual-delivered RRT dose in ICUs and its relationship with patient outcome, such as mortality and duration of mechanical ventilation and ICU stay. Our hypothesis was that a higher RRT dose would be associated with better patient outcomes.

## Materials and methods

This study was conducted from June 2005 to December 2007 in 757 patients enrolled in 30 ICUs in eight countries. The protocol was approved by the institutional review boards of the five Steering Committee members. Written informed consent was obtained from patients or next of kin when required by a centre's review board. The design of the study was published in 2005 [14], and registered in the Cochrane Renal Group (CRG110600093).

### Study population

**Figure 1:**
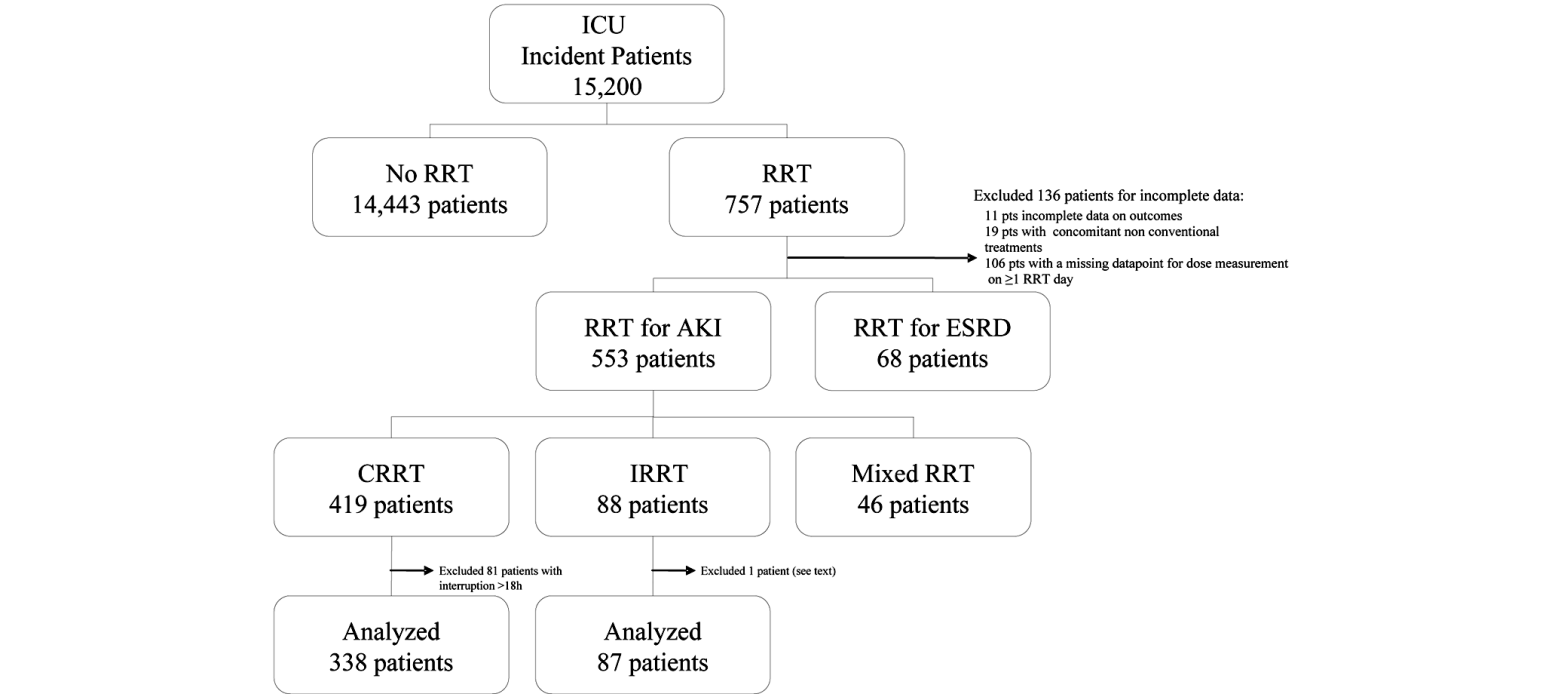
Profile of study population. Calculation of RRT dose was performed on patients who were treated exclusively on one RRT schedule (CRRT only or IRRT only). Forty six patients were treated with mixed RRT schedules (CRRT + CPFA, n = 10; CRRT + IRRT, n = 36; see text for explanation). AKI = acute kidney injury; CKD = chronic kidney disease; CPFA = coupled plasmafiltration adsorption; CRRT = continuous renal replacement therapy; ESRD = end-stage renal disease; ICU = intensive care unit; IRRT = intermittent renal replacement therapy.

All incident patients aged 12 years or older treated with RRT in the ICU were eligible for inclusion in the study. Patients with pre-existing chronic kidney disease stage 5 were excluded from analysis. Patients were categorised by treatment modality (Figure 1). AKI was defined using the Risk-Injury-Failure-Loss-End stage renal disease (RIFLE) classification [15].

### Data collection

Data from enrolled patients were entered into electronic case report forms resident on a password-protected web server [16]. Individual centres only had access to data relevant to their patients. Multiple data elements were collected for each patient [14]. Periodic audits were performed to establish the integrity of data capture and transfer into the database, as well as data accuracy.

### Calculation of delivered and prescribed RRT dose

Although several mathematical models have been developed to correlate the RRT dose given on different schedules (i.e. intermittent (IRRT) and continuous (CRRT)), none of these models have been rigorously validated in clinical practice [17-19]. We therefore chose to express the dose of CRRT and IRRT based on current clinical practice rather than a theoretical equivalent expression of dose [see Additional data file 1]. CVVH, continuous veno-venous haemodialysis (CVVHD), continuous veno-venous haemodiafiltration (CVVHDF) and high volume hemofiltration (HVHF) were analysed together as CRRT; dose was calculated using total effluent (the sum of the dialysate and ultrafiltrate) with correction for percentage predilution, and expressed as ml/kg/hour [20]. IRRT dose was expressed as the number of sessions per week [9]. Patients were categorised into those receiving more-intensive (CRRT ≥ 35 ml/kg/hour, IRRT ≥ 6 sessions/week) [9] or less-intensive (CRRT < 35 ml/kg/hour, IRRT < 6 sessions/week). Distribution of CRRT and IRRT dose are shown in Figure 1 in Additional data file 2. RRT and concurrent ICU care were instituted and prescribed at discretion of the treating physician.

### End points

ICU mortality was the primary outcome. The secondary outcomes were ICU length of stay and duration of mechanical ventilation.

### Statistical analyses

Continuous variables are expressed as mean ± standard deviation or median (interquartile range) and compared between any two groups using t-test or the Mann Whitney U test, and among three groups using analysis of variance (general linear models with adjustment for multiple comparisons) or the Kruskal-Wallis test, where appropriate. Categorical variables are expressed as proportions and compared with the Mantel-Haenszel chi-squares test or Fisher's exact test.

For the analysis of RRT dose versus outcome, CRRT and IRRT patients were analysed separately because of well-recognised differences between these populations in observational studies [21,22]. Exploratory univariate analysis for several variables was performed to identify possible risk (or protective) factors associated with ICU mortality. Multivariable logistic regression analysis was then conducted to test the relationship between RRT dose and ICU mortality, adjusted for confounding factors.

Based on the results of the univariate analysis, the covariates included in the CRRT model were sex, age (10-year increments), sequential organ failure assessment (SOFA) and serum creatinine at CRRT initiation and CRRT downtime (hours); for IRRT the covariates included were age (10-years increments), sex and RIFLE class at IRRT initiation. In addition to adjusting for significant covariates, residual confounding and selection effects were addressed using propensity scores. We generated a propensity score using multivariable logistic regression with more-intensive RRT dose as the dependent variable, as previously described [22,23]. Variables included in the propensity score were gender, weight, SOFA score and serum creatinine at RRT initiation. We fitted models for ICU mortality only adjusted for covariates and a combination of covariates plus the propensity score. We assessed for collinearity between variables using tolerance and variance inflation factors; there was no significant collinearity detected. The model's goodness of fit was tested with the Hosmer-Lemeshow statistic.

As sensitivity analyses, RRT dose was evaluated as both continuous variables and categorical variables. As continuous variable for CRRT, we used the actual value or as increments of 10 ml/kg/hour; for IRRT, we used number of IRRT sessions per week (possible range: 1 to 7). As categorical variables, we created RRT dose categories based on the literature, as well as standard statistical groupings (median, tertiles). Posthoc multivariate analyses were also performed limiting the analysis to specific subgroups of CRRT patients (septic patients, by simplified acute physiology score (SAPS) scores, ≥ 25 hours of CRRT). Because of the relatively small sample size of the IRRT, subgroup analysis was not performed.

Finally, ICU survival by RRT dose categories was presented graphically using Kaplan-Meier product limit survival plot. Two-tailed p values less than 0.05 were considered significant. Statistical analyses were conducted using STATA 10 (StataCorp LP, College Station, TX, USA).

## Results

### Enrollment and baseline characteristics

Characteristics of the participating centres are shown in Table 1 in Additional data file 2. The median enrollment period in each study centre was 384 days. During the study period, about 15,200 patients were admitted to participating ICUs. Among them, 757 patients were treated with RRT sometime during the ICU stay (Figure 1). Of the 757 enrolled patients, we excluded patients (n = 19) who received concomitant extracorporeal treatments (e.g. endotoxin adsorption) other than those specified in the methods of this report, and those with incomplete data (n = 117). The majority of incomplete data was due to one missing datapoint needed to calculate RRT dose on a specific day, such as percentage pre-dilution or actual start or stop time. Complete data on pre-specified outcomes were not available for 11 patients (1.4%).

**Table 1:** Clinical characteristics of ICU patients receiving RRT

	**All**	**CRRT**	**IRRT**	**Mixed RRT**	***P CRRT vs IRRT***	***P (three groups)***
n (%)	471	338 (72)	87 (18)	46 (10)		
Male sex (%)	67.7	66.6	70.1	71.7	0.529	0.679
Age (years)	63.6 ± 16.2	62.1 ± 16.9	68.3 ± 13	65.8 ± 14.7	0.004	0.009
Body weight (kg)	79.3 ± 19.3	79.4 ± 19.7	77.9 ± 19.6	81.4 ± 15	0.269	0.229
**ICU admission**						
SAPS II	50 ± 18	50 ± 17	50 ± 19	51 ± 21	0.508	0.788
SOFA	10 ± 4	10 ± 3	10 ± 3	11 ± 4	0.703	0.012
Creatinine (μmol/L)	99(80 to 150)	100 (80 to 150)	106 (80 to 177)	97 (80 to 115)	0.277	0.278
Chronic kidney disease (%)	56.3	55.9	55.2	60.9	0.901	0.796
**Diagnosis**						
Sepsis (%)	32.7	38.8	14.9	21.7	< 0.001	< 0.001
Post-surgical (%)	29.7	21.9	51.7	45.7	< 0.001	< 0.001
**Admission department**						
Emergency (%)	30.4	33.4	19.5	28.3	0.012	0.040
Medicine (%)	21.0	24.0	12.6	15.2	0.022	0.041
Surgery (%)	48.6	42.6	67.8	56.5	< 0.001	< 0.001
Hospital to ICU admission (days)	1 (0 to 5)	1 (0 to 4)	2 (0 to 7)	1 (0 to 5)	0.042	0.146
**RRT**						
ICU admission to start RRT (days)	3 (1 to 7)	2 (1 to 7)	3.5 (1 to 8.5)	3 (2 to 5)	0.884	0.700
RIFLE class at RRT initiation						
Risk (%)	11.3	12.7	5.8	10.9	0.067	0.186
Injury (%)	28.5	27.5	31.0	30.4	0.515	0.746
Failure (%)	57.3	56.8	60.9	54.3	0.489	0.759
Non-renal indication (%)	2.8	3.0	2.3	2.2	0.740	0.920
SOFA at RRT initiation	11 ± 3	12 ± 3	10 ± 2	13 ± 3	< 0.001	< 0.001
Creatinine at RRT initiation (μmol/L)	265 (177 to 368)	274 (177 to 380)	243 (203 to 301)	194 (141 to 309)	0.319	0.031
Start to end RRT (days)	3 (2 to 7)	3 (2 to 6)	4 (2 to 7)	13.5 (7 to 26.5)	0.113	< 0.001
**Indication for RRT initiation**						
Azotaemia	72.1	67.9	86.2	76.1	0.001	0.003
RIFLE class	64.4	64.9	60.9	67.4	0.495	0.715
Fluid overload	58.6	61.6	51.7	50.0	0.096	0.116
Oliguria	43.6	48.1	28.7	39.1	0.001	0.004
**Outcome**						
ICU mortality (%)	47.6	54.1	22.1	44.7	< 0.001	< 0.001
Mechanical ventilation (days)	10 (3 to 19)	10 (4 to 19)	8 (1 to 17)	16 (11 to 38)	0.037	0.002
ICU length of stay (days)	14 (7 to 27)	13 (6.5 to 26)	14 (6 to 23)	25 (15 to 42)	0.769	< 0.001

CRRT = continuous renal replacement therapy; ICU = intensive care unit; IRRT = intermittent renal replacement therapy; RIFLE = Risk-Injury-Failure-Loss-Endstage renal disease; RRT = renal replacement therapy; SAPS II = simplified acute physiology score; SOFA = sequential organ failure assessment.

Among the remaining 553 AKI patients, 419 received CRRT only, 88 received IRRT only and 46 were treated with mixed RRT schedules (IRRT, CRRT, coupled plasmafiltration adsorption). As patients in this last group crossed over from one RRT modality to another, delivered dose could not be calculated due to lack of clinically validated models, and they were excluded from the analysis. Among patients treated on only one RRT schedule (either continuous only or intermittent only), 82% received CRRT. This proportion represents current practice in Europe as previously reported. Out of 419 CRRT patients, 81 patients had at least one interruption of 18 hours or more, and then resumed CRRT [14]. The median interruption time was 49 hours (IQR = 29 to 113), predominantly due to filter clotting, disconnection for procedures and change in patient clinical status (e.g. CRRT not required in a window period). As daily CRRT dose would appear artificially low in this situation, such patients were not included in the analysis. Eighty eight patients (18%) were treated exclusively with IRRT. One patient had only three IRRT sessions over a span of 146 days, and was excluded from analysis (Figure 1).

Characteristics of the study population are described in Table 1. CRRT patients were younger, more likely to have sepsis, more likely to have been admitted directly into the ICU from the emergency room and less likely to be surgical patients. The mean serum creatinine at RRT initiation was 265 μmol/L. Nearly 60% of all patients were in RIFLE class Failure at RRT initiation. A small minority of patients did not meet criteria even for Risk, and were labelled as a non-renal indication (e.g. volume overload). Among the reasons cited to start RRT, azotaemia was significantly more common in the IRRT group, and oliguria in the CRRT group. Crude ICU mortality was 54% in the CRRT group, 22% in the IRRT group and 45% in the mixed group.

### Patient characteristics by RRT dose

#### CRRT

In the CRRT group, the median delivered RRT dose was 27.1 ml/kg/hour (IQR = 22.1 to 33.9). Only 75 patients (22%) received more-intensive dose (≥ 35 ml/kg/hour), while 262 (78%) received less-intensive CRRT. In further detail, 202 (60%) received a dose between 21 and 34 ml/kg/hour, and 61 (18%) received a dose of 20 ml/kg/hour or less (Table 2). Patients were also divided into tertiles of RRT dose (Table 2 in Additional data file 2). Median treatment downtime, i.e. the amount of time the CRRT was not running in a 24-hour period, was one hour (IQR = 0 to 2). The most common causes for CRRT interruption were clotting of the circuit (74% of episodes), vascular access problem (11%) and clinical reasons (10%). The median prescribed CRRT dose was estimated at 34.3 ml/kg/hour (IQR = 27.3 to 42.9). Eighty seven percent of patients used replacement fluid in various proportions of pre-dilution (median 70%, IQR = 33 to 100). Patients receiving CRRT of 35 ml/kg/hour or above had lower body weight, higher admission SAPS II and SOFA scores, and a trend towards lower serum creatinine at RRT initiation (Table 2). The net fluid removal did not differ between the more- and less-intensive groups: the median was 852 ml/day (IQR = 221 to 1693) in more-intensive CRRT, and 928 ml/day (IQR = 428 to 1996) in less-intensive CRRT (*P *= 0.22).

**Table 2:** Clinical characteristics of CRRT patients by CRRT dose (≤ 20, 21 to 34, and ≥ 35 ml/kg/hour)

	**Less-intensive (< 35 ml/kg/hour)**		**More-intensive**	
		
	**≤ 20 ml/kg/hour**	**21 to 34 ml/kg/hour**	**≥ 35 ml/kg/hour**	***P***
n (%)	61 (18)	202 (60)	75 (22)	
Male sex (%)	73.8	67.8	58.1	0.139
Age (years)	59.05 ± 19.0	63.48 ± 15.9	61.01 ± 17.4	0.226
Body weight (kg)	91.66 ± 24.4	79.70 ± 18.0	68.81 ± 12.8	< 0.001
**ICU admission**				
SAPS II	46 ± 19	51 ± 17	52 ± 16	0.030
SOFA	9 ± 4	10 ± 3	11 ± 4	0.030
Creatinine (μmol/L)	106 (88 to 150)	97 (80 to 150)	106 (80 to 150)	0.597
Chronic kidney disease (%)	47.5	58.9	55.4	0.290
**Diagnosis**				
Sepsis (%)	31.2	40.1	40.5	0.419
Post-surgical (%)	24.6	21.3	21.6	0.859
**Admission department**				
Emergency (%)	36.1	35.2	25.7	0.292
Medicine (%)	16.4	23.3	32.4	0.087
Surgery (%)	47.5	41.6	41.9	0.702
Hospital to ICU admission (days)	0.5 (0 to 4)	1 (0 to 4)	1 (0 to 3)	0.654
**RRT**				
ICU admission to RRT (days)	2.5 (2 to 8)	3 (2 to 7)	2 (1 to 3)	0.005
RIFLE class at RRT initiation				
Risk (%)	19.7	9.9	13.5	0.123
Injury (%)	18.0	27.7	35.1	0.086
Failure (%)	55.7	59.9	50.0	0.331
Non-renal indication (%)	6.6	2.5	1.4	0.168
SOFA at RRT initiation	11 ± 3	12 ± 3	12 ± 3	0.068
Creatinine at RRT initiation (μmol/L)	283 (177 to 389)	283 (194 to 415)	221 (168 to 327)	0.052
CRRT dose (ml/kg/hour)	15.4 ± 4.2	26.9 ± 4.0	44.8 ± 9.4	< 0.001
Start to end RRT (days)	3 (2 to 6)	4 (2 to 8)	2 (1 to 3)	< 0.001
**Indication for RRT initiation**				
Azotaemia	65.0	70.0	63.9	0.559
RIFLE class	56.7	70.0	56.9	0.048
Fluid overload	61.7	62.5	59.7	0.917
Oliguria	49.5	41.7	48.6	0.562
**Outcome**				
ICU mortality (%)	50.8	53.0	60.8	0.426
Mechanical ventilation (days)	13 (3 to 23)	12 (5 to 20)	5 (2.5 to 13)	< 0.001
ICU length of stay (days)	17 (7.5 to 29)	15 (9 to 27)	8 (4 to 18)	< 0.001

CRRT = continuous renal replacement therapy; ICU = intensive care unit; RIFLE = Risk-Injury-Failure-Loss-Endstage renal disease; RRT = renal replacement therapy; SAPS II = simplified acute physiology score; SOFA = sequential organ failure assessment.

#### IRRT

In the IRRT group (Table 3), the median delivered dose was 7 sessions/week (IQR = 5 to 7). Fifty six patients (64%) received more-intensive IRRT, while 31 (36%) were treated with the less-intensive dose. In further detail, 51 patients (59%) received daily dialysis, while five (6%) had 6 sessions/week, 10 (11%) had five sessions/week and 21 (24%) received alternate day dialysis (3 to 4 sessions/week). The median dialysis duration was 5.5 (IQR = 4 to 9) hours. The median prescribed Kt/V per session was estimated at 1.2 (IQR = 0.8 to 1.9). More-intensive IRRT patients were more likely to be septic compared with the less-intensive group (Table 3). The net fluid removal was similar between the more- and less-intensive groups: the median was 780 ml/day (IQR = 410 to 1115) in more-intensive IRRT, and 829 ml/day (IQR = 485 to 1103) in less-intensive IRRT (*P *= 0.68).

**Table 3:** Clinical characteristics of IRRT patients by IRRT dose (< 6 and ≥ 6 sessions per week)

	**Frequency < 6 days/week**	**Frequency ≥ 6 days/week**	***P***
n (%)	31 (36)	56 (64)	
Male sex (%)	71.0	69.6	0.897
Age (years)	69.13 ± 11.7	67.84 ± 13.7	0.873
Body weight (kg)	79.52 ± 15.7	76.99 ± 21.6	0.246
**ICU admission**			
SAPS II	49 ± 15	50 ± 20	0.958
SOFA	10 ± 3	9 ± 4	0.689
Creatinine (μmol/L)	106 (80 to 186)	106 (80 to 177)	0.822
Chronic kidney disease (%)	54.8	55.4	0.963
**Diagnosis**			
Sepsis (%)	3.2	21.4	0.023
Post-surgical (%)	64.5	44.6	0.076
**Admission department**			
Emergency (%)	12.9	23.2	0.245
Medicine (%)	12.9	12.5	0.957
Surgery (%)	74.2	64.3	0.343
Hospital to ICU admission (days)	3 (0 to 8)	1 (0 to 6)	0.322
**RRT**			
ICU admission to RRT (days)	5 (1 to 11)	3 (1 to 7)	0.351
RIFLE class at RRT initiation			
Risk (%)	6.5	5.4	0.834
Injury (%)	41.9	25.0	0.102
Failure (%)	51.6	66.1	0.186
Non renal indication (%)	0.0	3.6	0.287
SOFA at RRT initiation	10 ± 2	10 ± 2	0.941
Creatinine at RRT initiation (μmol/L)	247 (199 to 296)	234 (212 to 301)	0.880
Number of sessions per week	4.5 ± 0.8	6.9 ± 0.2	< 0.001
Start to end RRT (days)	6 (5 to 12)	3 (1 to 5)	< 0.001
**Indication for RRT initiation**			
Azotaemia	96.8	80.4	0.033
RIFLE class	58.1	62.5	0.685
Fluid overload	41.9	57.1	0.174
Oliguria	22.6	32.1	0.345
**Outcome**			
ICU mortality (%)	19.4	23.6	0.646
Mechanical ventilation (days)	14 (5 to 21)	6 (0 to 14)	0.030
ICU length of stay (days)	18 (13 to 31)	9.5 (6 to 18)	0.023

ICU = intensive care unit; IRRT = intermittent renal replacement therapy; RIFLE = Risk-Injury-Failure-Loss-Endstage renal disease; RRT = renal replacement therapy; SAPS II = simplified acute physiology score; SOFA = sequential organ failure assessment.

### Outcomes

#### CRRT

On univariate analysis, age, SAPS II, SOFA score and serum creatinine on admission, SOFA score and serum creatinine on RRT initiation, and RRT duration were significantly associated with ICU mortality. On multivariate analysis, CRRT dose was not associated with ICU mortality (Table 4). Further adjustment for the propensity score did not significantly alter this result (adjusted odds ratio (OR) = 1.40, 95% confidence interval (CI) = 0.74 to 2.65). Kaplan Meier curves are shown in Figures 2 and 3 in Additional data file 2. Results were similar whether CRRT dose was expressed as a continuous or categorical variable (Table 5). Additional post-hoc sensitivity analyses were performed (Table 6). Results were similar in patients with and without sepsis. We also performed subgroup analysis on the following patient subgroups: those with intermediate severity of illness (SAPS II scores from 45 to 60), and those who had a minimum exposure of 25 hours for CRRT therapy.

**Table 4:** Unadjusted and covariate adjusted analysis for ICU mortality in CRRT patients

**CRRT**	**Unadjusted analysis**			**Covariate adjusted analysis**		
	
	**OR**	**95% CI**	***P***	**OR**	**95% CI**	***P***
Male sex	1.47	0.91 to 2.37	0.097	1.86	1.11 to 3.12	0.019
Age (10-year increments)	1.34	1.17 to 1.52	< 0.001	1.42	1.22 to 1.64	< 0.001
SOFA at RRT initiation	1.18	1.09 to 1.28	< 0.001	1.20	1.10 to 1.30	< 0.001
Creatinine at RRT initiation (μmol/L)	0.85	0.76 to 0.95	0.005	0.79	0.69 to 0.90	0.001
Downtime	0.90	0.80 to 1.01	0.081	0.95	0.83 to 1.07	0.386
**More-intensive (≥ 35 ml/kg/hour)**	1.41	0.83 to 2.38	0.204	1.21	0.66 to 2.21	0.537

CI = confidence interval; CRRT = continuous renal replacement therapy; ICU = intensive care unit; OR = odds ratio; RRT = renal replacement therapy; SOFA = sequential organ failure assessment.

**Table 5:** Sensitivity analysis for ICU mortality in CRRT patients

**CRRT dose expressed as**	**Unadjusted OR with 95% CI**	***P *value**	**Adjusted OR with 95% CI**	***P *value**
Raw value (ml/kg/hour)	1.00 (0.98 to 1.02)	0.827	0.99 (0.97 to 1.02)	0.583
Increment of 10 ml/kg/hour	1.05 (0.87 to 1.26)	0.638	0.98 (0.79 to 1.23)	0.879
Literature cut-offs				
≤ 20 ml/kg/hour (ref.)	1.00		1.00	
21 to 34 ml/kg/hour	1.09 (0.61 to 1.93)	0.768	0.79 (0.40 to 1.55)	0.492
≥ 35 ml/kg/hour	1.50 (0.76 to 2.98)	0.245	1.00 (0.45 to 2.24)	0.995
Less-intensive (< 35 ml/kg/hour) (ref.)	1.00		1.00	
More-intensive (≥ 35 ml/kg/hour)	1.41 (0.83 to 2.38)	0.204	1.21 (0.66 to 2.21)	0.537
≤ 20 ml/kg/hour (ref.)				
> 20 ml/kg/hour	1.19 (0.68 to 2.07)	0.546	0.84 (0.43 to 1.61)	0.595
Tertiles				
1st tertile (≤ 23.5 ml/kg/hour) (ref.)	1.00		1.00	
2nd tertile (23.6 to 30.9 ml/kg/hour)	0.71 (0.42 to 1.20)	0.206	0.67 (0.37 to 1.23)	0.196
3rd tertile (≥ 31 ml/kg/hour)	1.32 (0.78 to 2.24)	0.306	1.11 (0.60 to 2.06)	0.734
1st+2nd tertiles (ref.)				
3rd tertile	1.56 (0.98 to 2.48)	0.058	1.36 (0.79 to 2.32)	0.268
Median				
< Median (ref.)	1.00		1.00	
≥ Median (≥ 27.1 ml/kg/hour)	0.99 (0.64 to 1.51)	0.945	0.82 (0.49 to 1.35)	0.433
CRRT dose in first 24 hours				
Less-intensive (< 35 ml/kg/hour) (ref.)	1.00		1.00	
More-intensive (≥ 35 ml/kg/hour)	1.02 (0.65 to 1.62)	0.918	0.96 (0.57 to 1.60)	0.866
CRRT dose in first 48 hours				
Less-intensive (< 35 ml/kg/hour) (ref.)	1.00		1.00	
More-intensive (≥ 35 ml/kg/hour)	1.08 (0.68 to 1.74)	0.737	1.03 (0.60 to 1.76)	0.915

CI = confidence interval; CRRT = continuous renal replacement therapy; ICU = intensive care unit; OR = odds ratio.

**Table 6:** Subgroup analysis for ICU mortality in CRRT patients

More-intensive vs less-intensive CRRT dose*	Unadjusted OR* with 95% CI	***P *value**	Adjusted OR* with 95% CI	***P *value**
*Patient subgroups*				
Sepsis	1.64 (0.70 to 3.85)	0.259	1.91 (0.71 to 5.13)	0.198
Non sepsis	1.27 (0.65 to 2.48)	0.488	0.95 (0.43 to 2.10)	0.896
SAPS II 45 to 60	1.03 (0.44 to 2.40)	0.945	0.67 (0.24 to 1.81)	0.428
SAPS II < 45 or > 60	1.69 (0.85 to 3.31)	0.129	1.76 (0.80 to 3.86)	0.159
≥ 25 hours of CRRT	1.06 (0.55 to 2.01)	0.870	1.07 (0.51 to 2.28)	0.855
< 25 hours of CRRT	1.72 (0.61 to 4.86)	0.303	1.12 (0.34 to 3.73)	0.854

*OR refers to more-intensive CRRT with respect to the reference group less-intensive CRRT.

CI = confidence interval; CRRT = continuous renal replacement therapy; ICU = intensive care unit; OR = odds ratio; SAPS II = simplified acute physiology score.

Similarly, there was no relation between CRRT dose and ICU mortality in these two subgroups. Patients who received more-intensive CRRT overall had shorter duration of mechanical ventilation and ICU stay (Table 7). Among survivors, there was a trend towards shorter ICU stay (*P *= 0.063), while duration of mechanical ventilation was significantly less (*P *= 0.031). Similar to the overall group, survivors in the more-intensive CRRT group had significantly lower body weight and shorter ICU stay prior to CRRT initiation (2 days, IQR = 1 to 3; vs. less-intensive 3 days, IQR = 2 to 7.5; *P *= 0.002), compared with the less-intensive CRRT group. Otherwise, the survivors in the two groups had similar baseline characteristics.

**Table 7:** ICU length of stay and ventilation days by RRT dose

	**Total**	**CRRT**		
		
		**< 35 ml/kg/hour**	**≥ 35 ml/kg/hour**	***P***
Length of ICU stay (days)	13 (6.5 to 26)	15 (9 to 28)	8 (4 to 18)	< 0.001
Patients who survived	19 (11 to 32)	19.5 (12 to 33.5)	15 (8 to 26)	0.063
Patients who died	10 (4 to 19)	12 (6 to 20)	4.5 (3 to 9.5)	< 0.001
				
Duration of MV (days)	10 (4 to 19)	12 (5 to 21)	5 (2.5 to 13)	< 0.001
Patients who survived	14 (4.5 to 22)	14 (5 to 24)	7 (4 to 17)	0.031
Patients who died	8.5 (3 to 17)	10 (5 to 18)	4 (2 to 9.5)	< 0.001

		**IRRT**		
		
	**Total**	**Frequency < 6 sessions/week**	**Frequency ≥ 6 sessions/week**	***P***

Length of ICU stay (days)	14 (6.5 to 23)	18 (15 to 31)	9.5 (6 to 18)	0.023
Patients who survived	11 (6 to 20)	18 (13 to 35)	8 (5.5 to 14)	0.008
Patients who died	17 (12 to 23)	18 (17 to 23)	15 (12 to 22)	0.597
				
Duration of MV (days)	8 (1 to 17)	14 (5 to 21)	6 (0 to 14)	0.030
Patients who survived	5 (0 to 13)	12 (3 to 24)	2.5 (0 to 10)	0.026
Patients who died	17 (11 to 21)	18 (17 to 21)	14 (8 to 18)	0.252

Data shown as median (interquartile range).

CRRT = continuous renal replacement therapy; ICU = intensive care unit; IRRT = intermittent renal replacement therapy; MV = mechanical ventilation.

#### IRRT

None of the variables examined, including IRRT dose, were significantly associated with ICU mortality on univariate and multivariate analysis (Table 8). This was seen whether IRRT dose was expressed as a dichotomous variable (more- vs. less-intensive) or as a continuous variable, i.e. number of sessions per week (unadjusted OR = 1.11, 95% CI = 0.74 to 1.69). Because of the relatively small sample size, no further sensitivity analysis was attempted. Patients who received more-intensive IRRT had shorter duration of mechanical ventilation and ICU stay, particularly among survivors (Table 7). Survivors in the more-intensive IRRT group were more likely to have sepsis (23% vs. less-intensive 0%, *P *= 0.008), compared with the less-intensive IRRT group. Otherwise, the survivors in the two groups had similar baseline characteristics.

**Table 8:** Unadjusted and covariate adjusted analysis for ICU mortality in IRRT patients

**IRRT**	**Unadjusted analysis**			**Covariate adjusted analysis**		
	
	**OR**	**95% CI**	***P***	**OR**	**95% CI**	***P***
Male sex	1.28	0.37 to 5.12	0.674	1.38	0.42 to 4.58	0.598
Age (10-year increments)	1.31	0.85 to 2.01	0.216	1.29	0.83 to 2.02	0.260
RIFLE class						
Risk (%)	2.51	0.19 to 23.49	0.320	1.00		
Injury (%)	2.45	0.74 to 7.92	0.089	1.29	0.2 to 8.33	0.790
Failure (%)	0.38	0.12 to 1.22	0.064	0.46	0.07 to 2.88	0.408
**More-intensive **(≥ 6 sessions/week)	1.29	0.43 to 3.82	0.646	1.50	0.48 to 4.67	0.482

CI = confidence interval; ICU = intensive care unit; IRRT = intermittent renal replacement therapy; OR = odds ratio; RIFLE = Risk-Injury-Failure-Loss-Endstage renal disease.

## Discussion

We conducted a European multicentre observational study to describe clinical outcomes associated with RRT dose in critically ill patients with AKI. The key findings of this study are the following. First, despite a prescribed CRRT dose approximating 35 ml/kg/hour, the recommended 'minimum' for critically ill AKI patients according to expert opinion [10,24], the delivered CRRT dose was markedly lower than this value. Second, it appears that alternate day IRRT for critically ill patients is uncommon in the participating centres. Third, after adjustment for multiple variables, we did not observe a beneficial effect of more-intensive RRT dose on ICU survival. Fourth, ICU stay and ventilation days were shorter in the more-intensive RRT groups.

Our findings on mortality are congruent with an international observational study [25] and two recent randomised clinical trials on standard versus higher-dose CVVHDF including the large multicentre Veterans Affairs/National Institute of Health (VA/NIH) trial in the US [8,9]. There are conflicting results on the effect of RRT dose on patient outcome. Two earlier single-centre randomised clinical trials showed a beneficial effect of an intensive CRRT dose when compared with a less-intensive dose in both CVVH (≥ 35 ml/kg/hour versus ≤ 20 ml/kg/hour) [4] and CVVHDF (≥ 42 ml/kg/hour versus ≤ 25 ml/kg/hour) [7]. In contrast, Bouman and colleagues did not detect any difference in outcome between ultrafiltration rates of 3 to 4 l/hour and 1 to 1.5 l/hour; however, this study suffered from lack of power and an unexpectedly high ICU survival rate among enrolled subjects [5]. Interestingly, in contrast to other studies [5,7-9], we observed a positive effect of more-intensive RRT dose on ICU stay and duration of mechanical ventilation.

Our study has several notable features. It is the first large observational study specifically oriented towards RRT dose involving multiple ICUs which are a mix of academic and non-academic centres. As such, it is likely to be more reflective of actual clinical practice and a broad patient population. Indeed, in the CRRT group, the prevalence of sepsis was 39% and the overall mortality was 54%. This is similar to that described in the literature [3,6,7,9,22].

Second, it focused on delivered, rather than prescribed, RRT dose. This is not a minor point, as many factors contribute to delivering a RRT dose lower than prescribed, and it is the delivered dose which the patient 'sees' and which is likely to affect the clinical outcome. A prior observational study reported only on prescribed, but not the delivered, CRRT dose [25]. Furthermore, ours is the first dose study in which correction for percentage predilution was performed in the calculation of CRRT dose, resulting in a more accurate estimate. It has been shown that delivered dialysis dose is generally lower than prescribed, ranging from 68 to 89% of prescribed [7,8,26,27].

Third, we specifically collected information on treatment 'downtime', which is considered an important factor affecting delivered RRT dose.

Fourth, it is also one of only two studies to look at a continuum of RRT dose so far [25]. Of note, the majority of patients received a CRRT dose which was in between the 'standard/low dose' and the 'high dose' arms evaluated in randomised clinical trials [4,7-9]. Whether having several patients in this 'intermediate' zone served to dilute the true clinical effect of RRT dose remains unclear. It has been suggested that only major changes in the application of dose can be reasonably expected to have a discernible clinical effect [28]. For example, the difference between a delivered CRRT dose of 30 and 35 ml/kg/hour may be too subtle, or could be criticised for being within a calculation error. However, we performed a variety of analyses which included expressing dose in increments of 10 ml/kg/hour (arbitrarily considered a 'significant' increment), or as categories based on cut-offs from the literature or on statistical spread (e.g. tertiles, median), and failed to find a significant effect on ICU mortality (Table 5). It is also possible that therapy in the first 24 to 48 hours is more crucial with respect to patient outcome. We therefore performed a post-hoc sensitivity analysis looking at CRRT dose during these periods (Table 5), and this did not significantly alter the results.

It has been suggested that septic patients may be a specific population which could benefit from higher RRT dose [4,11]. In our *post-hoc *analysis, the effect of RRT dose on mortality was similar in both septic and non-septic patients (Table 6). It is also possible that more-intensive RRT only benefits patients with an intermediate severity of illness, as suggested by Paganini and colleagues [29]. We performed two sensitivity analyses to address this. First, we limited the analysis only to patients with SAPS scores between 45 and 60, in whom the predicted mortality ranges from 35 to 60%. In five studies evaluating the effects of CRRT dose, mean acute physiology and chronic health evaluation (APACHE) II scores ranged from 22 to 26, giving predicted mortality rates of 42 to 57% in this group [4-9]. Our results were similar within this subgroup. Second, patients may have a very short duration of RRT for various reasons. For example, they may be gravely ill and die shortly after RRT initiation. Alternatively, they may be less ill and have rapid recovery of renal function allowing early withdrawal of RRT. Therefore, we performed a secondary analysis looking only at patients who had at least 25 hours of RRT. This was adapted from the definition of an 'adequate trial of therapy' in a randomised trial comparing CRRT and IRRT [30]. The results remained qualitatively unchanged.

This study provides further insight into the prescription and delivery of RRT dose in current clinical practice. There is a gap between prescribed and delivered CRRT dose, as has been shown by others [7,8,26,27]. Treatment downtime is a known contributing factor. In contrast to earlier studies, however, we also considered the effect of percentage pre-dilution in calculating the delivered dose. We hypothesise that lack of attention to this when prescribing CRRT may play a heretofore unrecognised role in under-delivery of dose. As modern machines are able to provide replacement fluid in variable proportions of pre/post-dilution, it is important to keep this in mind. We also observed that CRRT patients receiving more-intensive dose had significantly lower body weights. This may represent indiscriminate 'by the litre' prescription, rather than 'individualised' prescription based on body weight [13]. It is also possible that it is simply more difficult to provide higher doses in larger patients with currently available technology. Perhaps the greatest concern arising from the observed gap between prescription and delivery is the potential downstream effect of prescribing 20 ml/kg/hour to patients. There would be a real risk of effectively underdialysing patients [31].

We acknowledge certain limitations in this study. As with all observational studies, ours may have suffered from 'selection by prognosis' [32]. Indeed, it is quite plausible that, based on existing literature, the treating intensivist or nephrologist would prescribe a higher RRT dose to a sicker patient, who *a priori *has a higher predicted mortality. Although we adjusted for potential confounders, including propensity score analysis (Table 3 in Additional data file 2), this may still be insufficient because it is not possible to adjust for confounders that are neither measured nor known. We also chose to exclude a number of patients from the analysis, which may have resulted in some selection bias. It was not possible to analyse patients who crossed over between modalities because there is no single equivalent expression of dose clinically validated for both CRRT and IRRT. However, when we compared the analysed group to the overall population, they were similar in terms of demographics, general severity of illness and co-morbidities. Therefore, if selection bias was present, its effect is likely to be minimal. For IRRT patients, we were unable to correlate outcome with the measured Kt/V, as the necessary laboratory parameters for the calculation were not collected as part of routine practice. This is consistent with the findings of the VA/NIH group in their pre-trial survey that assessment of the delivered dose of IRRT was performed infrequently in clinical practice [13]. Nevertheless, we believe we have a reasonable estimate of prescribed IRRT dose based on the operational parameters collected, with a median prescribed Kt/V of 1.2. Although we found an inverse relationship between RRT dose and duration of mechanical ventilation, as well as with ICU stay, we acknowledge that there were no standard criteria for extubation or ICU discharge in this observational study. Furthermore, we only looked at short-term outcomes. Future studies should attempt to better understand the long-term effects of RRT dose. Lastly, this was a voluntary survey conducted in predominantly CRRT-oriented centres. As such, it may not be possible to generalise the results to other medical centres. It is noteworthy, however, that despite being CRRT-oriented centres, the delivered CRRT dose, although higher than reported in prior studies [26,28], still fell short of the mark. This begs the question as to whether a dose of 35 ml/kg/hour or 45 ml/kg/hour [4,11], as suggested for septic patients, is routinely achievable in the real world.

Nevertheless, our findings of reduced ICU stay and mechanical ventilation days with more-intensive RRT may potentially have a large impact on health resource utilisation, if confirmed by future studies. For example, the average total cost per ICU day has been estimated at €1200 in a sample of European countries [33]. A possible implication would be potential savings of €8000 to 10,200 per ICU admission with more-intensive RRT. In contrast to our results, two other studies showed no significant difference in duration of mechanical ventilation between lower and higher CRRT dose groups [5,7]; however, this issue was not specifically addressed by others [4,6,8,9].

This study highlights that the concept of RRT dose or adequacy is more complex than previously thought. This adds fuel to the debate on the optimal RRT dose for critically ill patients with AKI. Clearly there are other dimensions to RRT adequacy other than removal of various solutes, whether expressed as Kt/V, ml/kg/hour or number of RRT sessions per week. These include prophylactic volume control, as well as acid-base and tonicity control, among others. Furthermore, recognising that critical illness is not a static condition, a 'dynamic approach' to RRT dose, rather than fixed dose, may be more appropriate in this setting [34]. This hypothesis is worthy of exploration in future studies. In addition, it is likely that there are multifaceted interactions between RRT dose and other factors (timing of RRT, modality, patient characteristics, etc.) which influence outcome.

## Conclusions

We conducted a prospective European multicentre cohort study of AKI patients treated with RRT. This study provides insight in to how RRT is currently practiced in the ICU. We observed that the median CRRT dose is lower than 35 ml/kg/hour and only 22% of patients received this or a higher dose. In contrast, 60% of IRRT patients were treated daily. We evaluated the association between actual delivered RRT dose and clinical outcomes. The data provide no evidence for a survival benefit afforded by more-intensive RRT. However, higher RRT dose appeared to be associated with shorter ICU stay and duration of mechanical ventilation. In conclusion, within the confines of the dose range examined, there was no effect on survival while effects on non-mortality endpoints should be examined by further study.

## Key messages

• In this observational study, the delivered CRRT dose was markedly lower than 35 ml/kg/hour (median = 27).

• Alternate day IRRT for critically ill patients was uncommon in the participating centres.

• After adjustment for multiple variables, there was no beneficial effect of more-intensive RRT dose on ICU survival.

• Shorter ICU stay and duration of mechanical ventilation were observed in the more-intensive RRT groups.

## Abbreviations

AKI: acute kidney injury; APACHE: acute physiology and chronic health evaluation; CI: confidence interval; CRRT: continuous renal replacement therapy; CVVH: continuous veno-venous haemofiltration; CVVHD: continuous veno-venous haemodialysis; CVVHDF: continuous veno-venous haemodiafiltration; ICU: intensive care unit; IRRT: intermittent renal replacement therapy; IQR: interquartile range; RIFLE: Risk-Injury-Failure-Loss-Endstage renal disease; RRT: renal replacement therapy; SAPS II: simplified acute physiology score; SOFA: sequential organ failure assessment.

## Competing interests

The authors declare that they have no competing interests.

## Authors' contributions

SV participated in conception and design, patient enrollment, acquisition of data, analysis and interpretation of data, and critical revision of the manuscript. DNC participated in patient enrollment, acquisition of data, performed statistical analysis, analysis and interpretation of data, drafting and critical revision of the manuscript. RF, DKM, GM, AM, FM, MF, MM, RR and SL participated in conception and design, patient enrollment, acquisition of data and critical revision of the manuscript. CR conceived and designed the study, participated in analysis and interpretation of data, critical revision of the manuscript, and obtaining administrative and technical support.

## Supplementary Material

Additional data file 1 A Word file containing a more detailed description of the study methodology. Click here for file

Additional data file 2 A Word file containing three tables and three figures as listed. Table 1: Characteristics of participating centres. Table 2: Clinical characteristics of continuous renal replacement therapy (CRRT) patients by tertiles of RRT dose. Table 3: Unadjusted, covariate adjusted, and covariate + propensity score-adjusted analysis for intensive care unit (ICU) mortality in CRRT patients. Figure 1: Distribution of RRT dose by RRT modality. **(a) **CRRT, **(b) **intermittent RRT; Figure 2: Kaplan Meier curve for ICU survival by tertiles of CRRT dose; Figure 3: Kaplan Meier curve for ICU survival by CRRT dose (≤ 20, 21 to 34, and ≥ 35 ml/kg/hour). Click here for file
