# Supplementary material for: Delivered dose of renal replacement therapy and mortality in critically ill patients with acute kidney injury
Source: Crit Care. 2009 Apr 15;13(2):R57. doi: 10.1186/cc7784 (PMC2689504; doi:10.1186/cc7784)
Supplement: Additional data file 1 — A Word file containing a more detailed description of the study methodology. [file cc7784-S1.doc]

**Additional Data file 1 for:**

**Delivered Dose of Renal Replacement Therapy and Mortality in Critically Ill Patients with Acute Kidney Injury**

**Methods**

This study was conducted from June 2005 to December 2007 in 757 patients enrolled in 30 ICUs in 8 countries. The protocol was approved by the institutional review boards of the five Steering Committee members. Informed, written consent was collected from patients or next of kin when required by a center’s review board. The design of the study was published in 2005 [14], and registered in the Cochrane Renal Group (CRG110600093).

*Study population*

All incident patients with age ≥12 years, treated with RRT in the ICU were eligible for inclusion in the study. Patients with pre-existing CKD stage 5 were excluded from analysis. Patients were categorized by treatment modality (Figure 1). AKI was defined using the Risk-Injury-Failure-Loss-End stage renal disease (RIFLE) classification [15].

*Data Collection*

Data from enrolled patients were entered into electronic case report forms resident on a password-protected webserver [16]. Centers could only have access to data relevant to their patients. Multiple data elements were collected for each patient, including demographics, admission and discharge data, indications for RRT were collected. Blood chemistry was evaluated on a daily basis until termination of RRT. All-cause ICU mortality was recorded.

The case report forms and the data management strategy were previously described in detail (1). Data were centrally downloaded from the server in an Excel-based data collection tool (Microsoft Corp, Seattle, WA). Periodic audits were performed to establish the accuracy of data capture and transfer into the database. Four independent (2 information technology specialists and 2 nephrologists) regularly screened data for missing or out-of-range values and data inconsistencies. An email and/or telephone contact was made to the participating investigators requesting completion, correction, or verification of specific data items. Edit messages are retained in the file indicating the questionable data and any changes made when the requested information is returned. The final data were entered an Access file (Microsoft Corp, Seattle, WA) which was then imported into a statistical program for analysis.

*RRT and concurrent ICU care*

RRT was performed using volumetrically controlled, bicarbonate-based machines and synthetic biocompatible hemodialyzers. Continuous techniques included continuous veno-venous hemofiltration (CVVH), continuous veno-venous hemodialysis (CVVHD), and continuous veno-venous hemodiafiltration (CVVHDF), high volume hemofiltration (HVHF) and coupled plasma filtration and adsorption (CPFA) as previously defined [14]. Operational characteristics for both CRRT and IRRT were determined by the treating intensivists or nephrologists at each clinical site, as well as cross-over to other modalities. There were no pre-specified criteria for initiation or withdrawal of RRT or for any of its aspects, but detailed information was collected. Severity of AKI at RRT initiation was described using the Risk-Injury-Failure-Loss-Endstage renal disease (RIFLE) classification [15]. Concurrent ICU care for each patient was determined by the treating physicians, and no interventions were instituted as part of the DoReMi study.

*Calculation of delivered RRT dose*

Although several mathematical models have been developed to correlate the RRT dose given on different schedules (i.e. intermittent and continuous), none of these models have been rigorously validated in clinical practice[17-19]. We therefore chose to express the delivered dose of CRRT and IRRT as based on current clinical practice rather than a theoretical equivalent expression of dose, as was done in the VA/NIH trial [9]. CVVH, CVVHD, CVVHDF and HVHF were analyzed together as CRRT. The delivered CRRT dose was calculated using total effluent (the sum of the dialysate and ultrafiltrate) with correction for % predilution, utilizing the methods described and validated by Ricci *et al*, and expressed as ml/kg/hr [20]. The calculator works on the assumption that the urea sieving coefficient is 1 for convective therapies and that complete saturation of spent dialysate occurs during continuous dialysis. For IRRT, the delivered dose is ideally measured using specimens for pre- and an equilibrated post-dialysis urea. However, this was not possible in this observational study since post-dialysis urea specimens are rarely done during routine clinical practice. Therefore delivered dose in IRRT was expressed as the number of sessions per week as follows:

IRRT dose (sessions per week) = 7*(number of IRRT sessions/total duration of IRRT)

Where total duration of IRRT is the number of days from the first to the last IRRT session. This simulates the dose expression of IRRT used in the VA/NIH Acute renal failure Trial Network (ATN) study on intensive versus conventional renal support [9].

*Estimation of prescribed RRT dose*

We estimated the prescribed CRRT dose as follows:

Prescribed CRRT dose (ml/kg/hr) = (Qr + Qd)/ patient weight

Where Qr and Qd are expressed in ml/hr and weight in kg. This estimate does not take into consideration the effects of pre-dilution for replacement fluid nor net patient fluid removal [20]. The prescribed Kt/V for each IRRT session was estimated by standard methods using dialyzer characteristics, blood and dialysate flow rates, achieved net fluid removal and actual duration of treatment [35,36].

*End points*

ICU mortality was the primary outcome. The secondary outcomes were ICU length of stay and duration of mechanical ventilation.

*Statistical analyses*

Continuous variables are expressed as mean ± standard deviation or median (interquartile range) and compared between any two groups using t-test or the Mann Whitney U test, and among three groups using analysis of variance (general linear models with adjustment for multiple comparisons) or the Kruskal-Wallis test, where appropriate. Categorical variables are expressed as proportions and compared with the Mantel-Haenszel Chi-square test or Fisher exact test.

For the analysis of dose versus outcome, CRRT and IRRT patients were analyzed separately because of well-recognized differences between these populations in observational studies. Exploratory univariate analysis for several variables was performed to identify possible risk (or protective) factors associated with ICU mortality. Multivariable logistic regression analysis was then conducted to test the relationship between RRT dose and ICU mortality, adjusted for confounding factors. Based on the results of the univariate analysis, the covariates included in the CRRT model were sex, age (10-year increments), SOFA and serum creatinine at CRRT initiation and CRRT downtime (hours); for IRRT the covariates included were age (10-years increments), sex and RIFLE class at IRRT initiation. In addition to adjusting for significant covariates, residual confounding and selection effects were addressed using propensity scores. We generated a propensity score using multivariable logistic regression with more-intensive RRT dose as dependent variable, as previously described [21-23]. Variables included in the propensity score were gender, weight, SOFA score, and serum creatinine at RRT initiation. We fitted models for ICU mortality adjusted for covariates only and a combination of covariates plus the propensity score. We assessed for collinearity between variables using tolerance and variance inflation factors; there was no significant collinearity detected. The model’s goodness of fit was tested with the Hosmer-Lemeshow statistic.

As sensitivity analyses, RRT dose was evaluated as both continuous variables and categorical variables. As continuous variable for CRRT, we used the actual value or as increments of 10 ml/kg/hr; for IRRT, we used number of IRRT sessions per week (possible range: 1-7 ). As categorical variables, we created RRT dose categories based on the literature, as well as standard statistical groupings (median, tertiles). Posthoc multivariate analyses were also performed limiting the analysis to specific subgroups of CRRT patients (septic patients, by SAPS scores, ≥25 hrs of CRRT). Because of the relatively small sample size of the IRRT, subgroup analysis was not performed.

Finally, ICU survival by RRT dose categories was presented graphically using Kaplan-Meier product limit survival plot. Two-tailed p values < 0.05 were considered significant. Statistical analyses were conducted using STATA 10 (StataCorp LP, College Station, TX, USA).
