# Supplementary material for: Delivered dose of renal replacement therapy and mortality in critically ill patients with acute kidney injury
Source: Crit Care. 2009 Apr 15;13(2):R57. doi: 10.1186/cc7784 (PMC2689504; doi:10.1186/cc7784)
Supplement: Additional data file 2 — A Word file containing three tables and three figures as listed. Table 1: Characteristics of participating centres. Table 2: Clinical characteristics of continuous renal replacement therapy (CRRT) patients by tertiles of RRT dose. Table 3: Unadjusted, covariate adjusted, and covariate + propensity score-adjusted analysis for intensive care unit (ICU) mortality in CRRT patients. Figure 1: Distribution of RRT dose by RRT modality. (a) CRRT, (b) intermittent RRT; Figure 2: Kaplan Meier curve for ICU survival by tertiles of CRRT dose; Figure 3: Kaplan Meier curve for ICU survival by CRRT dose (≤ 20, 21 to 34, and ≥ 35 ml/kg/hour). [file cc7784-S2.doc]

**Additional Data file 2 for:**

**Delivered Dose of Renal Replacement Therapy and Mortality in Critically Ill Patients with Acute Kidney Injury**

**Supplementary Figures and Tables**

Table 1: Characteristics of participating centers

Table 2: Clinical characteristics of CRRT patients by tertiles of RRT dose

Table 3: Unadjusted, covariate adjusted, and covariate + propensity score-adjusted analysis for ICU mortality in CRRT patients

Figure 1: Distribution of RRT dose by RRT modality; Panel A: CRRT, Panel B: IRRT

Figure 2: Kaplan Meier curve for ICU survival by tertiles of CRRT dose

Figure 3: Kaplan Meier curve for ICU survival by CRRT dose (≤20, 21-34, and ≥35 ml/kg/hr)

Supplementary Table S1: Characteristics of participating centers

| **Type of Hospital** |  |  |
| --- | --- | --- |
| University Hospital or  affiliated to a University | 8 |  |
| Large urban | 16 |  |
| Small urban | 6 |  |
| **No of beds** |  |  |
| <500 | 7 |  |
| 500-999 | 17 |  |
| >1000 | 6 |  |
| **Type of ICU** |  |  |
| General | 26 |  |
| Surgical | 3 |  |
| Trauma and Burns | 1 |  |
| **No of beds** |  |  |
| <10 | 4 |  |
| 10-29 | 21 |  |
| >30 | 5 |  |
| **Total patients/ICU/year**  **(median last 2 years)** | 531 | (130-2938) |
| **AKI cases/year** | 63 | (20-165) |
| **Patients in need of RRT** | 32 | (15-80) |
| **RRT mode available** |  |  |
| CRRT | 27 |  |
| IRRT | 1 |  |
| CRRT + IRRT | 2 |  |

Supplementary Table S2: Clinical characteristics of CRRT patients by tertiles of RRT dose

|  | **CRRT tertiles** | | | |
| --- | --- | --- | --- | --- |
|  | **Lowest** | **Middle** | **Highest** | **p** |
| N (%) | 113 (33.4) | 112 (33.1) | 113 (33.4) |  |
| Male sex (%) | 73.5 | 70.5 | 56.3 | 0.014 |
| Age (y) | 61.81±16.5 | 63.28±16.7 | 61.31±17.6 | 0.545 |
| Body weight (kg) | 90.63±22.3 | 78.05±16.7 | 69.64±13.0 | <0.001 |
| **ICU Admission** |  |  |  |  |
| SAPS II | 49±19 | 52±17 | 51±17 | 0.198 |
| SOFA | 9±4 | 10±3 | 10±4 | 0.197 |
| Creatinine (μmol/L) | 106(80-168) | 88(80-124) | 106(78-152) | 0.132 |
| CKD (%) | 50.4 | 65.2 | 52.7 | 0.056 |
| **Diagnosis** |  |  |  |  |
| Sepsis (%) | 34.5 | 43.8 | 37.5 | 0.349 |
| Post-surgical (%) | 21.2 | 20.5 | 24.1 | 0.791 |
| **Admission department** |  |  |  |  |
| Emergency (%) | 37.2 | 36.6 | 25.9 | 0.130 |
| Medicine (%) | 20.4 | 21.4 | 30.4 | 0.157 |
| Surgery (%) | 42.5 | 42.0 | 43.8 | 0.962 |
| Hospital to ICU admission (days) | 0.5(0-4) | 1(0-5) | 1(0-3) | 0.585 |
| **RRT** |  |  |  |  |
| ICU admission to RRT (days) | 3(2-8) | 2.5(1-7) | 2(1-5) | 0.098 |
| RIFLE class at RRT initiation |  |  |  |  |
| Risk (%) | 17.7 | 6.3 | 13.4 | 0.032 |
| Injury (%) | 24.8 | 25.9 | 32.1 | 0.413 |
| Failure (%) | 54.0 | 64.3 | 52.7 | 0.157 |
| Non renal indication (%) | 3.5 | 3.6 | 1.8 | 0.666 |
| SOFA at RRT initiation | 11±3 | 11±3 | 12±3 | 0.114 |
| Creatinine at RRT initiation (μmol/L) | 277(177-389) | 283(199-437) | 239(168-327) | 0.086 |
| CRRT dose (ml/kg/hr) | 18.4±4.6 | 27.1±2.1 | 40.8±9.5 | <0.001 |
| **Indication for RRT initiation** |  |  |  |  |
| Azotemia | 66.1 | 70.9 | 66.4 | 0.689 |
| RIFLE class | 62.5 | 67.3 | 64.5 | 0.757 |
| Fluid overload | 59.8 | 66.4 | 59.1 | 0.473 |
| Oliguria | 44.6 | 50.9 | 48.2 | 0.644 |
| **Outcome** |  |  |  |  |
| ICU mortality (%) | 54.9 | 46.4 | 61.6 | 0.073 |
| Mechanical ventilation (days) | 13(5-22) | 13(5-24) | 6(3-13) | <0.001 |
| ICU length of stay (days) | 16(8.5-28.5) | 15(9-29) | 10(4-19) | <0.001 |

S Table 3: Unadjusted, covariate adjusted, and covariate + propensity score-adjusted analysis for ICU mortality in CRRT patients

| **CRRT** | **Unadjusted analysis** | | | **Covariate adjusted analysis** | | | **Covariate + propensity score adjusted analysis** | | |
| --- | --- | --- | --- | --- | --- | --- | --- | --- | --- |
|  | **OR** | **CI 95%** | **p** | **OR** | **CI 95%** | **p** | **OR** | **CI 95%** | **p** |
| Male sex | 1.47 | 0.91-2.37 | 0.097 | 1.86 | 1.11-3.12 | 0.019 | 1.73 | 1.02-2.93 | 0.041 |
| Age (10-yr increments) | 1.34 | 1.17-1.52 | <0.001 | 1.42 | 1.22-1.64 | <0.001 | 1.41 | 1.22-1.64 | <0.001 |
| SOFA at RRT initiation | 1.18 | 1.09-1.28 | <0.001 | 1.2 | 1.10-1.30 | <0.001 | 1.22 | 1.11-1.33 | <0.001 |
| Creatinine at RRT initiation (μmol/L) | 0.85 | 0.76-0.95 | 0.005 | 0.79 | 0.69-0.90 | 0.001 | 0.76 | 0.66-0.88 | <0.001 |
| Downtime | 0.9 | 0.80-1.01 | 0.081 | 0.95 | 0.83-1.07 | 0.386 | 0.93 | 0.92-1.06 | 0.278 |
| **More-intensive ( ≥35 ml/kg/hr)** | 1.41 | 0.83-2.38 | 0.204 | 1.21 | 0.66-2.21 | 0.537 | 1.40 | 0.74-2.65 | 0.299 |

Supplementary Figure 1: Distribution of RRT dose by RRT modality; Panel A: CRRT, Panel B: IRRT

**Panel A: CRRT**

**Panel B: IRRT**

Supplementary Figure 2: Kaplan Meier curve for ICU survival by tertiles of CRRT dose

Supplementary Figure 3: Kaplan Meier curve for ICU survival by CRRT dose (≤20, 21-34, and ≥35 ml/kg/hr)
